# The N Termini of TAR DNA-Binding Protein 43 (TDP43) C-Terminal Fragments Influence Degradation, Aggregation Propensity, and Morphology

**DOI:** 10.1128/MCB.00243-18

**Published:** 2018-09-14

**Authors:** Yasar Arfat T. Kasu, Samrawit Alemu, Angela Lamari, Nicole Loew, Christopher S. Brower

**Affiliations:** a Department of Biology, Texas Woman's University, Denton, Texas, USA; b Texas Academy of Mathematics & Science, Denton, Texas, USA

**Keywords:** TAR DNA-binding protein, aggregation, amyotrophic lateral sclerosis, proteasome, autophagy

## Abstract

Fragments of the TAR DNA-binding protein 43 (TDP43) are major components of intracellular aggregates associated with amyotrophic lateral sclerosis and frontotemporal dementia. A variety of C-terminal fragments (CTFs) exist, with distinct N termini; however, little is known regarding their differences in metabolism and aggregation dynamics. Previously, we found that specific CTFs accumulate in the absence of the Arg/N-end rule pathway of the ubiquitin proteasome system (UPS) and that their degradation requires arginyl-tRNA protein transferase 1 (ATE1). Here, we examined two specific CTFs of TDP43 (TDP43^219^ and TDP43^247^), which are ∼85% identical and differ at their N termini by 28 amino acids. We found that TDP43^247^ is degraded primarily by the Arg/N-end rule pathway, whereas degradation of TDP43^219^ continues in the absence of ATE1. These fragments also differ in their aggregation propensities and form morphologically distinct aggregates. This work reveals that the N termini of otherwise similar CTFs have profound effects on fragment behavior and may influence clinical outcomes in neurodegeneration associated with aggregation.

## Abstract (precis)

Fragments of the TAR DNA-binding protein 43 (TDP43) are major components of intracellular aggregates associated with amyotrophic lateral sclerosis and frontotemporal dementia. A variety of C-terminal fragments (CTFs) exist, with distinct N termini; however, little is known regarding their differences in metabolism and aggregation dynamics.

## INTRODUCTION

**FIG 1:**
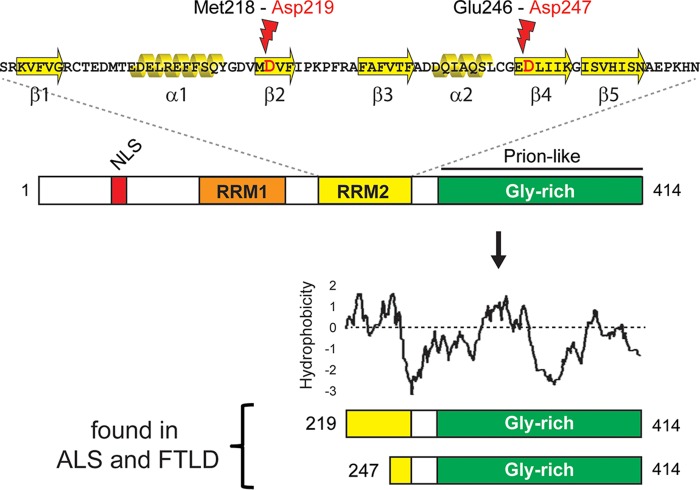
Features of TDP43 and two of its C-terminal fragments associated with neurodegeneration. (Top) Primary and secondary structures of human TDP43 RNA recognition motif 2 with locations of endoproteolytic cleavage occurring between Met218 and Asp219 and between Glu246 and Asp247 under conditions associated with amyotrophic lateral sclerosis (ALS) and frontal temporal lobar dementia (FTLD) (37). (Middle) Human TDP43 consists of 414 amino acids and contains a tandem set of RNA recognition motifs (RRM1 and RRM2) as well as a nuclear localization signal (NLS) and a glycine-rich C-terminal domain that includes a prion-like region. (Bottom) TDP43^219^ and TDP43^247^ generated by the cleavages shown at the top. Also shown is a Kyte-Doolittle plot showing the relative hydrophobicity throughout the fragments. The window size is 9.

The TAR DNA-binding protein 43 (TDP43) is a DNA- and RNA-binding protein originally identified through its ability to bind to the HIV type 1 TAR DNA sequence motifs and later shown to play a role in mRNA splicing, transport, and translational regulation, including the formation of stress granules (1–13). Although found primarily in the nucleus due to a nuclear localization signal (NLS) near its N terminus, TDP43 shuttles between the nucleus and the cytoplasm (14). TDP43 also contains a glycine-rich C terminus that includes a prion-like domain important for interaction with a number of RNA-binding proteins (Fig. 1) (14–17). TDP43 binds to UG-rich regions in the 3′ untranslated regions (UTRs) of mRNAs through tandem RNA recognition motifs (RRMs) that adopt a typical RRM architecture consisting of a β-sheet made up of antiparallel β-strands, stacked on two α-helix motifs (15). Although both RRMs are required for high-affinity RNA binding, each one is capable of binding nucleic acid individually (15). RRM2 is unique in that it contains an additional β-strand (β4) that forms a number of intramolecular contacts when bound to RNA and intermolecular contacts in the absence of RNA (15). The β4-strand also forms part of a cryptic nuclear export signal that becomes exposed in C-terminal fragments (CTFs) following proteolytic cleavage resulting from pathological conditions, including oxidative stress (18, 19), endoplasmic reticulum (ER) stress (20, 21), thermal stress (22), loss of RNA binding (23), or acetylation (24). Numerous studies have shown that CTFs of TDP43 form toxic, insoluble, cytoplasmic aggregates in patients with amyotrophic lateral sclerosis (ALS) and frontotemporal lobar dementia (FTLD) (16, 23, 25–30). The link between TDP43 and neurodegeneration is further strengthened by a number of disease-related mutations found within the C-terminal domain of TDP43 (31) and the occurrence of TDP43-related proteinopathies for other neurodegenerative disorders as well, including Alzheimer's disease (32, 33). Despite its strong association with neurodegenerative disease, it is unclear if cytotoxicity results from a loss of normal TDP43 function (e.g., mRNA processing) due to cleavage or from a toxic gain of function resulting from the accumulation and aggregation of CTFs. CTFs can apparently also act in a dominant negative fashion by nucleating cytoplasmic inclusions that include full-length TDP43 (23, 30, 34–38).

Although soluble TDP43 is thought to be degraded primarily by the ubiquitin (Ub) proteasome system (UPS), autophagy is thought to be required for the removal of insoluble aggregates (39–41). Intracellular protein homeostasis is established by both the UPS and autophagy, and defects in either contribute to a number of mammalian diseases (42–47). Previous work has shown an age-related decline in the ability of cells to maintain proper protein homeostasis, and a number of neurodegenerative disorders have been associated with decreased autophagy (48, 49) or UPS activity (50–54). The regulated degradation of protein fragments is achieved through a specific UPS pathway called the Arg/N-end rule pathway (55–57). Fragments bearing N-terminal basic amino acids (Arg, Lys, or His) or bulky hydrophobic amino acids (Phe, Leu, Trp, Tyr, or Ile) can be directly recognized by the Ub ligases of the N-end rule pathway (Ubr1, Ubr2, Ubr4, and Ubr5), which facilitate their polyubiquitylation and subsequent degradation by the proteasome. N-terminal Val, Gly, Pro, Ser, Thr, and Met (if not followed by a bulky hydrophobic residue [58]) are not recognized by the Arg/N-end rule pathway and are therefore nondestabilizing (55, 56). N-terminal Asn, Gln, Asp, Glu, and Cys are also destabilizing but require enzymatic modifications, including deamidation of Asn and Gln by Ntan1 and Ntaq1, respectively (59, 60), and N-terminal arginylation of Asp, Glu, or oxidized Cys by the *Ate1*-encoded arginyl-transferase (arginyl-tRNA protein transferase 1 [ATE1]) (61–63). ATE1-dependent posttranslational arginylation of proteins is emerging as an important player in maintaining nervous system function and in preventing neurodegeneration (64–68).

Previous studies using protein chemical analysis determined that the predominant TDP43-derived CTFs in cytoplasmic inclusions isolated from the brains of patients suffering from ALS and FTLD initiate with Arg^208^, Asp^219^, or Asp^247^ (37, 69, 70). Previously, we showed that these CTFs were short-lived substrates of the Arg/N-end rule pathway and that those bearing N-terminal Asp (TDP43^219^ and TDP43^247^) require ATE1 for their degradation by the N-end rule pathway (65). Here, we compared TDP43^219^ and TDP43^247^, which are ∼85% identical and differ by a hydrophobic N terminus of 28 amino acids (Fig. 1), and found that they have different fates in the absence of ATE1. While TDP43^247^ is degraded primarily by the Arg/N-end rule pathway in a manner that requires ATE1, an additional UPS pathway(s) is capable of degrading TDP43^219^ in the absence of ATE1. Consequently, in contrast to TDP43^247^, which forms aggregates in the majority of ATE1-lacking cells, TDP43^219^ has a low aggregation propensity in the absence of ATE1 and, under conditions that promote its accumulation, assembles into morphologically distinct aggregates. This work provides evidence that relatively small differences in the N termini of otherwise similar aggregation-prone fragments can have profound effects on fragment metabolization, aggregation propensity, and morphology and may influence clinical outcomes in neurodegeneration.

## RESULTS

### TDP43^219^ and TDP43^247^ are differentially degraded in the absence of ATE1.

**FIG 2:**
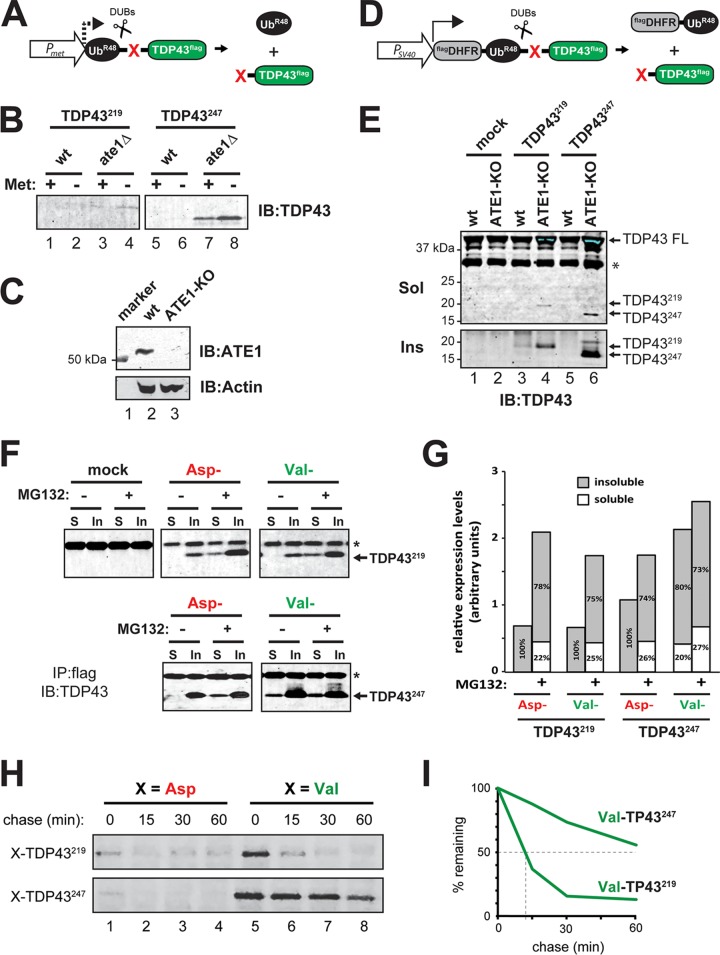
Differential degradation of TDP43^219^ and TDP43^247^ in the absence of ATE1 and the Arg/N-end rule pathway. (A) TDP43^219^ and TDP43^247^ were expressed in yeast by using the ubiquitin fusion technique (71). Cotranslational cleavage by cellular deubiquitylases (DUBs) yields Ub^R48^ (which contains a K48R mutation to prevent its participation in polyubiquitin chains that direct proteins to the proteasome for degradation) and a test fragment (TDP43^219^ or TDP43^247^) bearing a specified N-terminal amino acid (red X) and a C-terminal FLAG epitope tag. The *P_M_*_*et*_ promoter was used to induce expression when grown in methionine-lacking medium. (B) SDS-PAGE and immunoblotting (IB) using an anti-TDP43 antibody to detect steady-state levels of TDP43^219^ and TDP43^247^ expressed in wild-type (wt) and ATE1-lacking (*ate1*Δ) yeast. Expression was induced by growth in medium lacking methionine (Met). (C) ATE1 is detected by immunoblotting at ∼55 kDa in lysates of wild-type N2a cells but not in N2a cells that have undergone CRISPR/Cas9-mediated *ATE1* ablation. The bottom panel shows actin used as a loading control. (D) The ubiquitin reference technique (URT), derived from the Ub fusion technique (71). Cotranslational cleavage by DUBs of a URT-based fusion produces, at an initially equimolar ratio, a stable internal reference protein such as ^FLAG^DHFR-Ub^R48^, a FLAG-tagged derivative of the mouse dihydrofolate reductase, and a C-terminally FLAG-tagged test fragment (TDP43^219^ or TDP43^247^) bearing a specified N-terminal amino acid (red X). Moderate expression in mammalian cells was achieved by using the *P_SV40_* promoter. (E) Steady-state levels of TDP43^219^ and TDP43^247^ expressed in wild-type N2a cells and those that had undergone CRISPR-mediated knockout of *ATE1* (ATE1-KO). Full-length (FL), endogenous mouse TDP43 as well as exogenously added TDP43^219^ and TDP43^247^ were detected in detergent-soluble (Sol) and urea-soluble (Ins) fractions by SDS-PAGE and immunoblotting using an anti-TDP43 antibody. (F) Partitioning of detergent-soluble and -insoluble TDP43^219^ and TDP43^247^ bearing N-terminal Asp or N-terminal Val (not arginylated by ATE1), which were expressed in HEK293T cells. Cells were treated with 5 μM MG132 for 24 h, as indicated, prior to lysis. Fragments were immunoprecipitated (IP) from the soluble (S) and insoluble (In) fractions using an anti-FLAG antibody and detected by immunoblotting using an anti-TDP43 antibody. Asterisks represent the antibody light chain. (G) Graphical representation of the densitometric analysis of bands in panel F to show relative expression levels and the percentages of the total found in the soluble or insoluble fraction. (H) Pulse-chase analysis of TDP43^219^ and TDP43^247^ bearing either N-terminal Asp or Val in HEK293T cells. Fragments were labeled with [^35^S]Met-Cys, followed by a chase, preparation of extracts, immunoprecipitation with anti-FLAG, SDS-PAGE, and autoradiography. (I) Quantification of N-terminal Val-bearing fragments in panel H.

To confirm the role of ATE1 in the degradation of CTFs of TDP43, we compared the steady-state levels of TDP43^219^ and TDP43^247^ in the presence and absence of ATE1 in yeast (Saccharomyces cerevisiae) as well as in a murine neuroblastoma cell line (N2a). In yeast, the inducible *P_Met_* promoter was used to induce the expression of TDP43^219^ and TDP43^247^ in wild-type and *ate1*Δ yeast strains when grown in medium lacking methionine. CTFs were expressed as Ub fusions (Fig. 2A) so that cotranslational cleavage by cellular deubiquitylases would produce TDP43^219^ or TDP43^247^ bearing its natural N-terminal Asp amino acid (71). Neither CTF was detected in wild-type extracts, indicating that they are degraded to negligible levels at steady state. In extracts from *ate1*Δ yeast, however, TDP43^219^ and (especially) TDP43^247^ were stabilized (Fig. 2B). We saw a similar pattern when TDP43^219^ and TDP43^247^ were expressed in wild-type N2a cells and N2a cells that had undergone CRISPR/Cas9-mediated knockout of the endogenous *ATE1* gene (ATE1-KO cells) (Fig. 2C). At steady state, TDP43^219^ and TDP43^247^ (both bearing N-terminal Asp) were not detected in extracts from wild-type N2a cells but were readily detected in extracts from ATE1-KO cells (Fig. 2E). Consistent with numerous studies showing that aggregation-prone CTFs of TDP43 form insoluble proteinaceous inclusions in the cytoplasm, the bulk of TDP43^219^ and TDP43^247^ detected in ATE1-KO cells was found in the detergent-insoluble fractions (Fig. 2E, bottom). We also detected a slight increase in the amount of endogenous, full-length mouse TDP43 in the detergent-soluble fraction (conditions which do not lyse the nucleus) in ATE1-KO cells (Fig. 2E, lanes 4 and 6), which is consistent with previous reports that TDP43-derived CTFs are capable of seeding cytoplasmic aggregates containing mislocalized, full-length TDP43 (72, 73).

In order to investigate the effects of the Arg/N-end rule pathway and the UPS on CTF partitioning between the detergent-soluble and -insoluble fractions, TDP43^219^ and TDP43^247^ were expressed in HEK293T cells (which have robust N-end rule activity) bearing either N-terminal Asp or N-terminal Val (which is not arginylated by ATE1) in the presence or absence of the proteasome inhibitor MG132 for 24 h. Soluble and insoluble fractions were prepared, and CTFs were detected by anti-FLAG immunoprecipitation followed by SDS-PAGE and immunoblotting using an anti-TDP43 antibody (Fig. 2F). Similar to their metabolism in yeast and in N2a cells, TDP43^219^ and TDP43^247^ bearing their natural N-terminal Asp were not detected in soluble fractions of HEK293T cells but were detected in the insoluble fractions (Fig. 2F and G), indicating that CTFs that are not normally degraded by the proteasome ultimately form insoluble aggregates. The presence of MG132 resulted not only in a significant increase in the total amount of CTFs detected (∼3-fold and ∼1.6-fold for Asp-TDP43^219^ and Asp-TDP43^247^, respectively) but also in a significant shift in partitioning to the soluble fraction (∼22% and ∼26% of the total for Asp-TDP43^219^ and Asp-TDP43^247^, respectively) (Fig. 2F and G). N-terminal Val, which is not recognized by ATE1, had different effects on the overall levels and the partitioning of TDP43^219^ compared to TDP43^247^. In the absence of MG132, the overall levels of Val-TDP43^247^ increased 1.6-fold relative to those of Asp-TDP43^247^, and ∼20% of the total Val-TDP43^247^ was detected in the soluble fraction. MG132 did not significantly change the overall levels of Val-TDP43^247^ or its relative partitioning, suggesting that TDP43^247^ is degraded primarily by the Arg/N-end rule pathway. In contrast, Val-TDP43^219^ is not detected in the soluble fraction unless MG132 is present, indicating that soluble TDP43^219^ is degraded by the UPS even in the absence of its recognition by ATE1 and degradation by the Arg/N-end rule pathway.

In order to determine the fate of newly formed TDP43 CTFs, we utilized the ubiquitin reference technique (URT) to carry out *in vivo* pulse-chase analysis in HEK293T cells (71). URT-based fusion proteins generate, at initially equimolar concentrations, a stable reference protein (^FLAG^DHFR-Ub^R48^) and CTFs bearing specified N-terminal amino acids (Fig. 2D). Using this method, the fates of [^35^S]Met-Cys-labeled CTFs of TDP43 were monitored by measuring CTF levels relative to the level of ^FLAG^DHFR-Ub^R48^ at various time points after the label was washed out and translation was blocked by cycloheximide. Asp-TDP43^219^ and Asp-TDP43^247^ were detected at low levels even at the zero time point, consistent with our previous study showing that they are ATE1-dependent substrates of the Arg/N-end rule pathway (Fig. 2H) (65). The otherwise identical fragments bearing N-terminal Val, however, were degraded at different rates. The half-life of Val-TDP43^247^ extended beyond 60 min, whereas Val-TDP43^219^ was significantly less stable and degraded with a half-life of ∼10 min (Fig. 2H and I). These data indicate that soluble TDP43^247^ largely depends on the Arg/N-end rule pathway for its degradation, whereas an additional pathway(s) participates in the degradation of TDP43^219^.

### TDP43^219^ and TDP43^247^ differ in their propensities to form aggregates.

**FIG 3:**
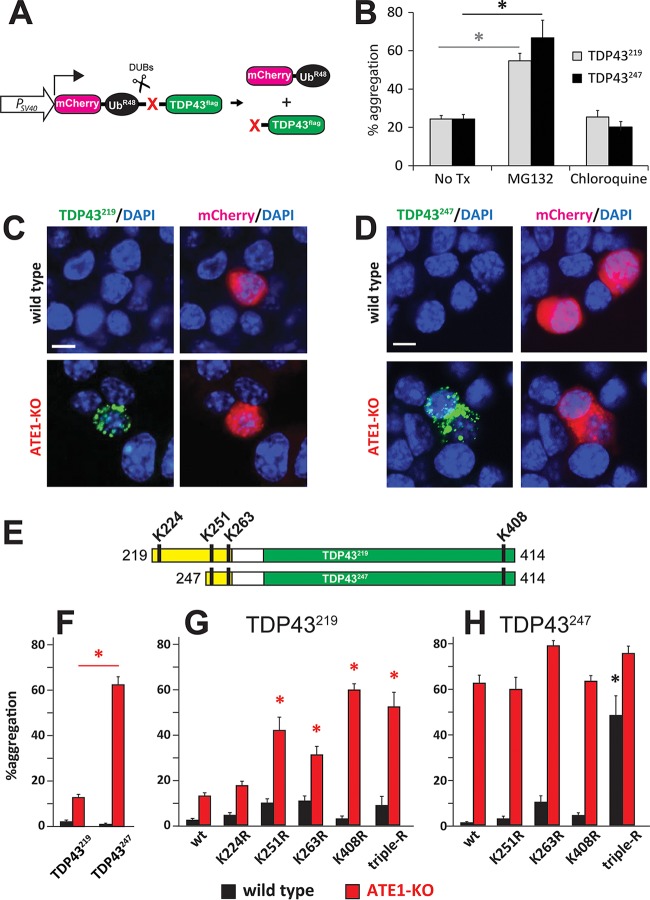
*In vivo* aggregation of TDP43^219^ and TDP43^247^. (A) Modified version of the URT whereby DHFR is replaced by mCherry in order to identify cells expressing aggregation-prone fragments. (B) HEK293T cells were transiently transfected with TDP43^219^ and TDP43^247^ and then treated with either 10 μM MG132 for 6 h or 50 μM chloroquine for 24 h prior to fixation. At 48 h posttransfection, cells were fixed, and aggregates were detected by using anti-FLAG primary antibody and an Alexa Fluor 488-conjugated secondary antibody. The percentages of mCherry-positive cells containing aggregates were quantified. Error bars indicate standard errors of the means (SEM). Statistical analyses were done by employing the unpaired *t* test. Asterisks represent bars that are significantly different from results with no treatment (No Tx) (*P* < 0.05). (C) Representative images of either wild-type (top) or ATE1-KO (bottom) mouse N2a cells transfected with a plasmid expressing mCherry-Ub^R48^-TDP43^219^ as seen in panel A. mCherry-Ub^R48^ was detected by red fluorescence, and TDP43^219^ was detected by indirect immunofluorescence, using anti-FLAG antibody and an Alexa Fluor 488-conjugated secondary antibody. Bars, 10 μm. (D) Similar to panel C except that cells were expressing TDP43^247^. (E) Schematic of TDP43^219^ and TDP43^247^ indicating relative positions of lysine residues. (F) Percentages of mCherry-positive wild-type and ATE1-KO N2a cells containing aggregates of TDP43^219^ or TDP43^247^. Error bars indicate SEM. Statistical analyses employed the unpaired *t* test (*P* = 0.0021). (G) Percentages of mCherry-positive wild-type and ATE1-KO N2a cells containing aggregates of wild-type TDP43^219^ or the indicated K→R mutations. (H) Same as panel G except with wild-type TDP43^247^ or the indicated K→R mutations. Error bars indicate SEM. Statistical analyses were done by using the unpaired *t* test. Asterisks represent bars that are significantly different from the wild-type fragment in the same cell type (*P* < 0.05).

Previous studies have shown that CTFs of TDP43 have a higher propensity to aggregate *in vivo* than full-length TDP43 (65, 74). In order to examine the relative aggregation propensities of TDP43^219^ and TDP43^247^ in cells, we used a modified version of the URT where the cDNA encoding dihydrofolate reductase (DHFR) was replaced with cDNA encoding the red-fluorescent mCherry protein (Fig. 3A). Cotranslational cleavage of this fusion construct yields stable mCherry-Ub^R48^, which “marks” transfected cells, and a C-terminally FLAG epitope-tagged fragment whose fate can be monitored by using indirect immunofluorescence with an anti-FLAG primary antibody and a fluorescein-conjugated secondary antibody (Fig. 3C and D).

Although our results using MG132 suggested that the UPS is involved in the ATE1-independent degradation of TDP43^219^ (Fig. 2F and G), we compared aggregate formation in HEK293T cells treated with either MG132 or chloroquine, a neutralizer of lysosomal pH which blocks degradation through autophagy. We found a far-greater increase in the aggregation propensities of both TDP43^219^ and TDP43^247^ as a result of MG132 treatment than with chloroquine treatment (Fig. 3B), suggesting that aggregation propensity is determined primarily by the UPS-mediated degradation of soluble CTFs.

In order to compare their relative aggregation propensities in the presence and absence of ATE1, TDP43^219^ and TDP43^247^ were expressed in both wild-type N2a cells and ATE1-KO N2a cells. Consistent with our previous finding that the Arg/N-end rule pathway can degrade CTFs of TDP43 (65), very few cells containing aggregates were detected in mCherry-positive, wild-type N2a cells. On the other hand, cytoplasmic, perinuclear aggregates of TDP43 CTFs could be detected in ATE1-KO cells (Fig. 3C and D). Interestingly, while aggregates of TDP43^247^ could be detected in ∼60% of mCherry-positive ATE1-KO cells, aggregates of TDP43^219^ could be detected in only ∼10% of mCherry-positive ATE1-KO cells (Fig. 3F). This is yet further evidence indicating that an additional pathway participates in the degradation of TDP43^219^ and, in doing so, prevents its aggregation even in the absence of ATE1.

TDP43^219^ and TDP43^247^ are identical throughout 85% of their sequences. However, TDP43^219^ possesses a hydrophobic, N-terminal, 28-amino-acid extension that includes a lysine residue at position 224 (relative to full-length human TDP43), which is not shared with TDP43^247^ (Fig. 3E). To determine if K224 played a role in aggregation propensity, we converted it to arginine (K224R), a conservative but nonubiquitylated residue, and examined its aggregation propensity in wild-type and ATE1-KO N2a cells. Surprisingly, the aggregation propensity of TDP43^219^-K224R in ATE1-KO N2a cells was not significantly increased, suggesting that K224 does not play a significant role in the UPS-mediated degradation of TDP43^219^ (Fig. 3G). The finding that TDP43^219^-K224R is not significantly more aggregated in wild-type cells also suggests that K224 is not a preferred site of ubiquitylation by the Arg/N-end rule pathway (Fig. 3G).

The amino acid sequence common to both TDP43^219^ and TDP43^247^ contains three additional lysine residues: K251 and K263 are located near the N terminus, and K408 is located at the extreme C terminus (Fig. 3E). In attempts to determine which lysine residues play an important role in the UPS-mediated degradation of CTFs and thereby prevent aggregate formation, they were each individually and collectively converted to arginine in both TDP43^219^ and TDP43^247^. Because TDP43^247^ is degraded primarily by the Arg/N-end rule pathway, no single mutation of any lysine residue or their collective mutation (TDP43^247^-tripleR) resulted in a significant increase in aggregation relative to nonmutated TDP43^247^ in ATE1-KO cells (Fig. 3H, red bars). In wild-type N2a cells, the aggregation propensity of TDP43^247^ should increase if a lysine normally ubiquitylated by the Arg/N-end rule pathway is mutated to arginine (Fig. 3H, black bars). Interestingly, mutation of K251, which is 6 residues from the N terminus of arginylated TDP43^247^, did not affect aggregation propensity, implying that K251 is sterically inaccessible to the ubiquitin ligases of the N-end rule of the Arg/N-end rule pathway. Although K263R had the greatest effect on the aggregation propensity of TDP43^247^, the effect was modest, suggesting that the Arg/N-end rule pathway is capable of ubiquitylating multiple lysine residues. This was substantiated by a dramatic increase in the aggregation of lysineless TDP43^247^-tripleR in wild-type N2a cells (Fig. 3H).

Although TDP43^219^ is capable of being degraded by the Arg/N-end rule pathway, our data indicate that an additional pathway, which is not capable of degrading TDP43^247^, participates in the degradation of TDP43^219^. To determine the relative contributions of the Arg/N-end rule pathway and the non-Arg/N-end rule pathway in the metabolism of TDP43^219^, we examined the aggregation propensity of TDP43^219^ harboring specific lysine mutations. In wild-type N2a cells, the K263R and K251R mutants had the greatest (albeit weak) effect on the aggregation propensity of TDP43^219^ (Fig. 3G, black bars). On the other hand, K408R had the greatest effect on the aggregation propensity of TDP43^219^ in ATE1-KO N2a cells (Fig. 3G, red bars). These results are consistent with the notion that the degradation of soluble TDP43^219^ is carried out predominantly by the Arg/N-end rule pathway, with a preference for ubiquitylation at K263 and/or K251, but in the absence of ATE1, an alternative UPS pathway, with a preference for K408, can facilitate its turnover. The finding that TDP43^247^, which also contains K408, is stable in the absence of ATE1 suggests that the 28-amino-acid N-terminal extension in TDP43^219^ harbors a degradation signal required for recognition by the alternative UPS pathway.

### Posttranslational modifications of TDP43^219^ and TDP43^247^.

**FIG 4:**
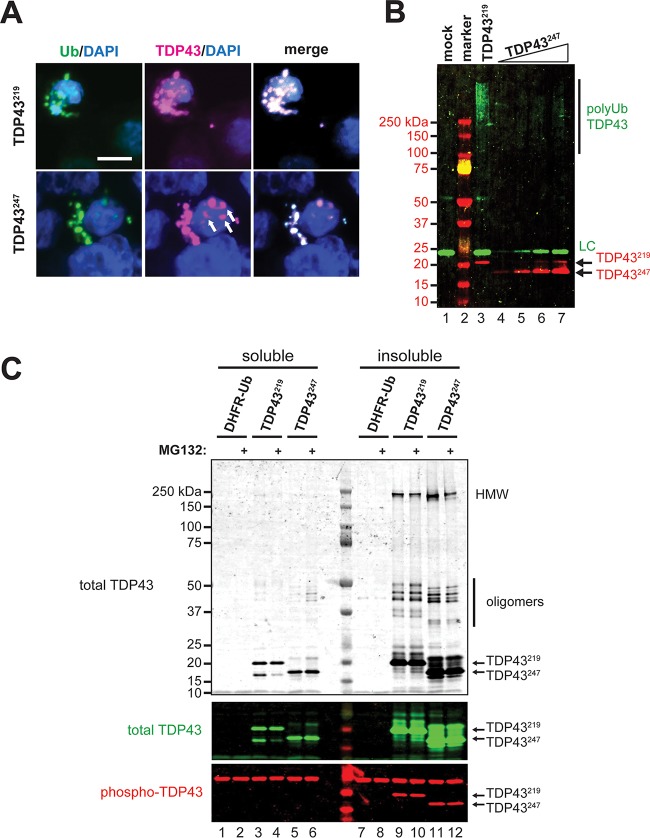
Posttranslational modifications of TDP43^219^, TDP43^247^, and their aggregates. (A) Immunocytochemistry reveals the colocalization of ubiquitin and Val-TDP43^219^ or Val-TDP43^247^ in aggregates formed in HEK293T cells treated with 5 μM MG132 for 24 h prior to fixation. Ubiquitin was detected by using an antiubiquitin antibody, and CTFs were detected by using an anti-FLAG antibody. Bar, 10 μm. (B) Relative polyubiquitylation of detergent-insoluble (urea-solubilized) Val-TDP43^219^ and Val-TDP43^247^ expressed in cells treated with 5 μM MG132 for 24 h. Polyubiquitylation was detected by immunoprecipitation of CTFs using an anti-FLAG antibody, followed by SDS-PAGE and immunoblotting using an antiubiquitin antibody. Note the increase in the polyubiquitylation of TDP43^219^ versus similar levels of TDP43^247^ (compare lanes 3 and 5). LC, antibody light chain. (C, top and middle) Monomeric, oligomeric, and high-molecular-weight (HMW) species of TDP43^219^ and TDP43^247^ were detected in detergent-insoluble fractions of HEK293T cells expressing CTFs by immunoprecipitation using an anti-FLAG antibody, followed by SDS-PAGE and immunoblotting using an anti-TDP43 antibody. (Bottom) Phosphorylated monomers of TDP43^219^ and TDP43^247^ were detected only in insoluble fractions. Phosphorylation of oligomers and high-molecular-weight species was not detected (not shown).

Aggregation-prone CTFs of TDP43 have been shown to be highly phosphorylated and ubiquitylated (29, 75, 76). We found that aggregates of TDP43^219^ completely colocalized with ubiquitin (Fig. 4A), whereas some TDP43^247^ aggregates either were not associated with ubiquitin or were associated to a lesser extent (Fig. 4A, arrows). In order to examine the relative associations of TDP43^219^ and TDP43^247^ with ubiquitin, Val-TDP43^219^ and Val-TDP43^247^ were expressed by using the URT (Fig. 2D), except that the highly expressing *P_CMV_* promoter was used and the UPS-mediated degradation was blocked by MG132 to promote accumulation and aggregation of CTFs. CTFs from the insoluble fractions were then isolated by immunoprecipitation using an anti-FLAG antibody, and ubiquitylation was evaluated by immunoblotting using an anti-Ub antibody. Ubiquitylation associated with insoluble CTFs were seen as a high-molecular-weight smear at >250 kDa (Fig. 4B). Interestingly, when samples were normalized for the amount of CTF, insoluble TDP43^219^ was ∼12-fold more heavily ubiquitylated than insoluble TDP43^247^ (Fig. 4B, compare lane 3 to lanes 4 through 7). The finding that Val-TDP43^247^ is less ubiquitylated than Val-TDP43^219^ is consistent with the existence of an additional UPS pathway capable of ubiquitylating TDP43^219^ but not TDP43^247^.

The bulk of soluble TDP43^219^ and TDP43^247^ was detected as monomers, whereas a significant portion of TDP43^219^ and TDP43^247^ isolated from insoluble fractions was recovered as oligomeric and high-molecular-weight species (∼250 kDa) that were resistant to urea as well as SDS-PAGE (Fig. 4C). Interestingly, we also found that soluble TDP43^219^ was processed (cleaved) into a fragment that migrated more similarly to TDP43^247^ in SDS-PAGE gels (Fig. 4C, lanes 3 and 4). Since our anti-TDP43 antibody recognizes epitopes in the C terminus of human TDP43, this species is a result of N-terminal cleavage of TDP43^219^. Remarkably, this processing was inhibited by MG132. In order to determine the phosphorylation status of TDP43^219^ and TDP43^247^, we carried out immunoblotting on both soluble and insoluble TDP43^219^ and TDP43^247^ using an anti-Ser409/410 phosphorylation-specific TDP43 antibody. Phosphorylated TDP43 was not detected in the soluble fractions (Fig. 4C, lanes 1 to 6); however, monomeric CTFs were phosphorylated in the insoluble fractions (Fig. 4C, lanes 7 to 12), consistent with phosphorylation playing a role in aggregation dynamics of insoluble TDP43 species (76).

### TDP43^219^ and TDP43^247^ form morphologically distinct cytoplasmic aggregates.

**FIG 5:**
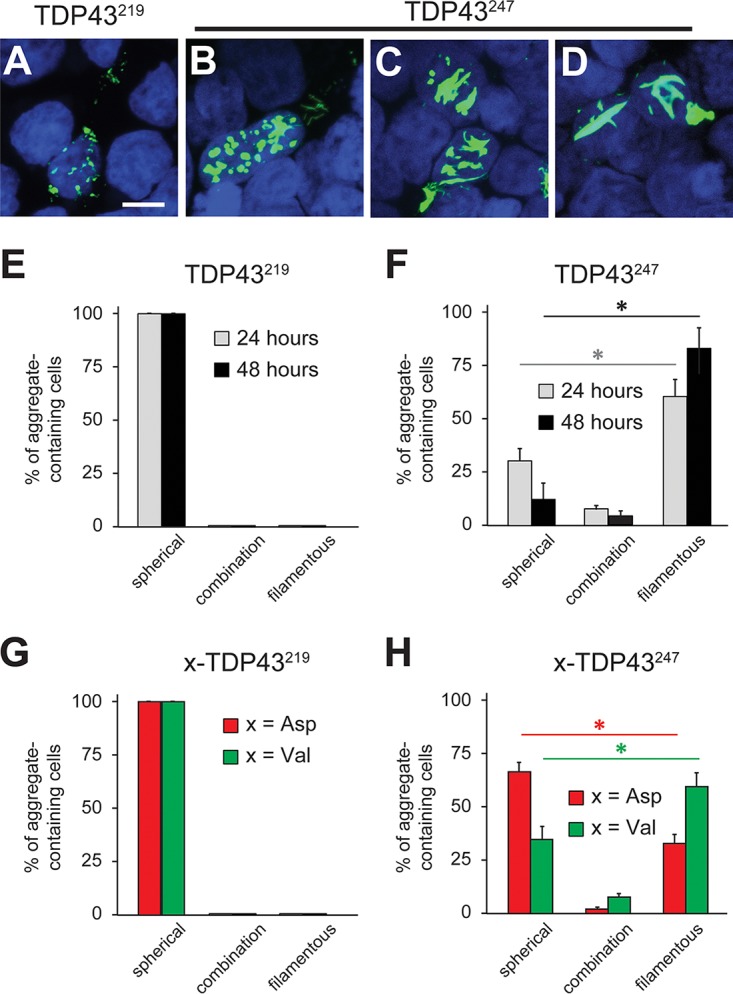
Aggregate morphology of TDP43^219^ and TDP43^247^. (A to D) Representative images of cytoplasmic aggregates formed in HEK293T cells by TDP43^219^ (A) and TDP43^247^ (B to D). Bar, 10 μm. (E) Quantification of spherical versus filamentous aggregates formed by Val-TDP43^219^ at 24 and 48 h posttransfection. (F) Same as panel E except that Val-TDP43^247^ was used. Error bars indicate SEM. Statistical analyses were done by employing the unpaired *t* test (*P* < 0.05) to compare spherical versus filamentous aggregates at 24 and 48 h. (G) Comparison of aggregate morphology with TDP43^219^ bearing N-terminal Asp versus N-terminal Val at 24 h posttransfection. Cells were treated with 10 μM MG132 for 6 h prior to fixation. (H) Same as panel G except that TDP43^247^ was used. Error bars indicate SEM. Statistical analyses were done by employing the unpaired *t* test (*P* < 0.05) to compare spherical versus filamentous aggregates formed by TDP43^247^ bearing N-terminal Asp and N-terminal Val.

Previous studies reported spherical (both large and small) and filamentous (skein-like) TDP43-containing inclusions in upper and lower motor neurons as well as in specific brain cell populations associated with ALS and FTLD (75, 77). Although TDP43^219^ aggregates were scarce relative to TDP43^247^ aggregates (Fig. 3F), we noticed that TDP43^219^ formed aggregates that were small and spherical (dot-like), whereas TDP43^247^ formed larger spherical and often filamentous aggregates (Fig. 5A to D). In order to determine if spherical aggregates mature into filamentous aggregates over time, we transfected HEK293T cells with Val-TDP43^219^ or Val-TDP43^247^ and quantified cells containing spherical, filamentous, or a combination of aggregates at 24 h and 48 h posttransfection. We were unable to detect filamentous aggregates of Val-TDP43^219^ at either time point (Fig. 5E) but found that the number of cells containing spherical TDP43^247^ aggregates decreased (∼30% versus ∼12.5% at 24 and 48 h, respectively) with a concomitant increase in filamentous TDP43^247^ aggregates over time (∼61.2% and ∼82.6% at 24 and 48 h, respectively) (Fig. 5F). In order to rule out the possibility that the nonnatural N-terminal Val residue influences aggregate morphology, we compared aggregate morphologies of TDP43^219^ and TDP43^247^ bearing either N-terminal Val or their natural N-terminal Asp at 24 h posttransfection (Fig. 5G and H). One possibility is that filamentous TDP43 aggregates form only after a critical concentration of soluble CTFs is reached. Since TDP43^247^ is degraded primarily by the Arg/N-end rule pathway, whereas TDP43^219^ is degraded by the Arg/N-end rule pathway and an additional UPS pathway (Fig. 2 and 3), steady-state levels of TDP43^219^, even in the absence of its degradation by the Arg/N-end rule pathway, may be insufficient to form filamentous aggregates. Therefore, we used MG132 to prevent all UPS-mediated degradation and promote the accumulation of CTFs (Fig. 2F and G). Even in the presence of MG132 and with high-level expression using the *P_CMV_* promoter, only small spherical aggregates were detected for both Asp-TDP43^219^ and Val-TDP43^219^ (Fig. 5G). On the other hand, both Asp-TDP43^247^ and Val-TDP43^247^ were capable of forming filamentous aggregates (Fig. 5H). Collectively, these results indicate that the N-terminal 28 amino acids of TDP43^219^ limit its aggregation morphology to smaller, dot-like, spherical aggregates, whereas TDP43^247^ is capable of forming spherical aggregates (which tended to be larger) as well as filamentous aggregates.

## DISCUSSION

In this study, we provide evidence that differences in the N termini of otherwise similar aggregation-prone C-terminal fragments of TDP43 can have a profound influence on fragment metabolism, on the propensity to form insoluble aggregates, and on aggregate morphology. We found that TDP43^247^ is degraded primarily by the Arg/N-end rule pathway in a manner that requires ATE1 but that an additional pathway(s) is capable of degrading TDP43^219^ even in the absence of ATE1. This is supported by multiple lines of evidence. First, steady-state levels of TDP43^219^ and TDP43^247^ in yeast and N2a cells lacking ATE1 show a greater accumulation of TDP43^247^ than of TDP43^219^ (Fig. 2B and E). Second, soluble Val-TDP43^247^, which is not recognized by ATE1 and therefore is not degraded by the Arg/N-end rule pathway, is stable, whereas soluble Val-TDP43^219^ is unstable unless the proteasome is inhibited (Fig. 2F and G). Third, experiments involving the metabolic labeling of CTFs reveal that in the absence of degradation by the Arg/N-end rule pathway, the half-life of TDP43^219^ is significantly shorter than that of TDP43^247^ (Fig. 2H and I). Fourth, aggregates of TDP43^247^ are detected in the majority of ATE1-lacking N2a cells, whereas aggregates of TDP43^219^ are detected in only ∼10% of ATE1-lacking N2a cells (Fig. 3F). Finally, insoluble TDP43^219^ is highly ubiquitylated, whereas insoluble TDP43^247^ is relatively free of ubiquitin (Fig. 4A and B). A number of proteins have been reported to play a role in TDP43 clearance (65, 78–80). Recently, the β4-strand of the RRM2 domain of human TDP43 was shown to harbor a degradation signal recognized by the VHL/ElonginB/C/Cul2/Rbx1 complex (80). Studies to identify the ATE1-independent UPS pathway contributing to the degradation of TDP43^219^ are under way.

We also found that despite being ∼85% similar, TDP43^219^ and TDP43^247^ form morphologically distinct intracellular aggregates (Fig. 5). Recently, Guenther et al. reported that the ^247^DLIIKGISVHI^257^ segment from RRM2 of TDP43 is capable of forming a range of amyloid polymorphs (81). We found that TDP43^219^ formed only small spherical aggregates, whereas TDP43^247^, which contains the ^247^DLIIKGISVHI^257^ segment directly at its N terminus, formed both spherical aggregates and filamentous aggregates over time (Fig. 5). The finding that TDP43^219^ does not form filamentous aggregates indicates that the additional N-terminal 28 amino acids (not included in TDP43^247^) function to prevent filamentous morphology. Previous studies analyzing the distribution of TDP43-immunoreactive neuronal inclusions in patients with ALS identified both spherical and skein-like inclusions in lower motor neurons of the spinal cord as well as in the motor cortex and the substantia nigra; however, TDP43-containing aggregates in the neostriatum and dentate gyrus were neither spherical nor skein-like (75, 77). In light of our findings, we suggest that distinct cleavages of TDP43 may occur in different neuronal populations under pathological conditions, resulting in fragment populations that differ in metabolism, aggregation propensity, and aggregate morphology, as determined (at least in part) by their N termini. Interestingly, Mori et al. detected spherical inclusions in motor neurons of ALS or FTLD patients with a disease duration of less than 100 months but only filamentous aggregates in motor neurons of patients with a disease duration of more than 100 months (77). Our data indicate that aggregates of at least some CTFs of TDP43 (e.g., TDP43^247^) undergo a time-dependent maturation process to form filamentous aggregates (Fig. 5F).

Multiple cleavage sites have been described for TDP43; therefore, a heterologous mixture of fragments can be generated under pathological conditions. Previous work has shown that TDP43 can be sequentially processed (21). As such, a given CTF can be a derivative of either full-length TDP43 or a larger “precursor” fragment. Of note, we detected a soluble CTF species resulting from N-terminal cleavage of TDP43^219^ migrating similarly to TDP43^247^ in SDS-PAGE gels (Fig. 4C). Although the protease responsible for this species is unknown, this cleavage is blocked by the proteasome inhibitor MG132 (Fig. 4C). It remains to be determined if this processing is catalyzed by the proteasome itself or by a separate cellular protease inhibited by MG132.

Collectively, our results suggest that cells may use N-terminal processing as a means to convert aggregation-prone fragments into species with altered properties. Such processing may alter the manner in which a fragment is degraded. If so, then the vulnerability of a given cell to the effects of a specific aggregation-prone fragment would be established by the complement of cellular proteases and the activity of particular degradation pathways within that cell. This may explain why some neuronal populations are more vulnerable than others to protein aggregates (e.g., Lewy bodies in neurons of the substantia nigra).

In sum, we found that the N termini of otherwise similar aggregation-prone C-terminal fragments of TDP43 have profound effects on its (i) metabolism, (ii) aggregation propensity, and (iii) aggregate morphology. This suggests that the N termini of aggregation-prone fragments may influence clinical outcomes in neurodegeneration. Ultimately, characterization of such differences may provide prognostic indicators useful in outcome predictions and may assist in the design of therapeutics for neurodegenerative diseases.

## MATERIALS AND METHODS

### Miscellaneous reagents.

Cycloheximide was obtained from Sigma-Aldrich. MG132 was obtained from Cayman Chemical. Chloroquine was obtained from Invitrogen/Thermo Scientific. Anti-C-terminal TDP43 (catalog number 12892-1-AP) and anti-phospho-TDP43 (catalog number 66318-1-Ig) were obtained from Proteintech. Anti-FLAG M2 (catalog number F1804) and anti-FLAG M2 magnetic beads (catalog number M8823) were obtained from Sigma-Aldrich. Anti-ATE1 (catalog number sc-398805), antiactin (catalog number sc-47778), and anti-ubiquitin P4D1 (catalog number sc-8017) were obtained from Santa Cruz Biotechnology. Detection was carried out by using the following goat secondary antibodies from Thermo Scientific: anti-mouse IgG(H+L)–Alexa Fluor 488 (catalog number A-11001), anti-rabbit IgG(H+L)–Alexa Fluor 546 (catalog number A11010), anti-mouse IgG–DyLight 680 (catalog number PI35519), anti-rabbit IgG–DyLight 680 (catalog number SA5-10176), anti-mouse IgG–DyLight 800 (catalog number PI35569), and anti-rabbit IgG–DyLight 680 (catalog number SA5-10036).

### Synthesis of plasmids.

To construct the plasmids pCB398 and pCB399 used in *in vivo* aggregation assays (Fig. 3), DNA encoding mCherry was amplified from pKP551 (see Table S1 in the supplemental material) by using primers CB383F and CB384R. The resulting PCR product was used to replace the Flag-DHFR moiety in pCB334 and pCB336, respectively, after digestion with HindIII/AgeI. The Flag-DHFR moiety in pCB340 was replaced with the mCherry cDNA to produce pCB401. For the generation of pCB541, pCB542, pCB543, and pCB544, Ub^K48R^-X-TDP43^219^ or Ub^K48R^-X-TDP43^247^ (where X is Asp or Val) was amplified by PCR and inserted into pRS416 (ATCC 87521) after digestion with HindIII/XhoI. For the generation of plasmids encoding K→R mutants of TDP43^219^ and TDP43^247^ (pYK002 to pYK016) as well as control vectors expressing mCherry-Ub^K48R^ only (pYK001) or ^FLAG^DHFR-Ub^K48R^ only (pYK027), the Q5 site-directed mutagenesis kit (New England BioLabs) was used according to the manufacturer's protocol. Briefly, plasmids were made by amplifying specific template plasmids with specific primer sets. Primer sets were designed to be nonoverlapping, and one primer contained specified mutations in the middle of the oligonucleotide sequence (Table S2). pYK001 was made by amplifying pCB400 with primers YK006R and YK007F. pYK004 was made by amplifying pCB398 with primers YK009R and YK010F. pYK005 was made by amplifying pCB398 with primers YK013F and YK014R. pYK006 was made by amplifying pCB398 with primers YK015F and YK016R. pYK007 was made by amplifying pCB398 with primers YK017F and YK018R. pYK009 was made by amplifying pYK007 with primers YK013F and YK014R. pYK010 was made by amplifying pCB400 with primers YK017F and YK018R. pYK011 was made by amplifying pYK005 with primers YK025F and YK027R. pYK012 was made by amplifying pYK006 with primers YK026F and YK027R. pYK015 was made by amplifying pYK009 with primers YK015F and YK016R. pYK016 was made by amplifying pYK015 with primers YK025F and YK027R. pYK027 was made by amplifying pCB328 with primers YK027R and YK032F. PCR was carried out for 25 cycles, and 1 μl of the resulting reaction mix was then used in a kinase-ligase-DpnI (KLD) reaction for 5 min at room temperature to obtain ligation and template removal. NEB5α competent cells (New England BioLabs) were transformed with 5 μl of the KLD reaction mixture and selected on LB plates supplemented with ampicillin (100 μg/ml).

### Expression of TDP43^219^ and TDP43^247^ in S. cerevisiae.

S. cerevisiae JD52 (*MAT***a** *trp1*-Δ*63 ura3-52 his3*-Δ*200 leu2-3112 lys2-801*) and CHY21 (*ate1*Δ::KanMX6 in JD52) were described previously (65). S. cerevisiae media included YPD medium (1% yeast extract, 2% peptone, and 2% glucose; only the most relevant medium components are cited), SD medium (0.17% yeast nitrogen base, 0.5% ammonium sulfate, 2% glucose), and synthetic complete (SC) medium (0.17% yeast nitrogen base, 0.5% ammonium sulfate, and 2% glucose plus a dropout mixture of compounds required by auxotrophic strains). S. cerevisiae was transformed with the low-copy-number pCB541, pCB542, pCB543, or pCB544 plasmid by using standard techniques (82). Expression of TDP43^219^ and TDP43^247^ was induced by growth in synthetic defined medium (lacking Ura and Met) for 24 h at 30°C. Equivalent numbers of yeast cells were resuspended in protein-loading buffer (PLB) (80 mM Tris [pH 6.8], 2% SDS, 10% glycerol, 0.0006% bromophenol blue, and 0.1 M dithiothreitol [DTT]) and lysed by sonication. The resulting samples were centrifuged at 13,000 rpm at room temperature, and proteins from the supernatant were boiled at 95°C and separated by SDS–4 to 15% PAGE followed by transfer to a polyvinylidene difluoride (PVDF) membrane (Bio-Rad) in Towbin buffer (25 mM Tris, 192 mM glycine, and 20% methanol). Membranes were blocked in phosphate-buffered saline (PBS) containing 5% milk and 0.1% Tween 20 at room temperature for 1 h. Primary anti-TDP43 C-terminal antibody and goat anti-rabbit secondary antibody were used to detect TDP43^219^ and TDP43^247^. Blots were developed by using the Licor Odyssey CLx system.

### Cell culture and transfection.

Human embryonic kidney HEK293T cells and neuroblastoma (N2a) cells were maintained at 37°C with 5% CO_2_ in Dulbecco modified Eagle medium (DMEM) (Corning Cellgro) containing 15% fetal bovine serum (Gemini Bio-products) supplemented with 20 mM glutamine, 100 U/ml penicillin, and 0.1 mg/ml streptomycin (Fischer Bioreagents). Cells were transfected by using the BioT transfection reagent (Bioland Scientific) according to the manufacturer's protocol.

### Generation of ATE1-lacking N2a cells.

Mouse neuroblastoma cells (N2a) were generated by using the CRISPR/Cas9 system (83). Briefly, pCB431, a plasmid encoding human codon-optimized Cas9 derived from Streptococcus pyogenes and a chimeric guide RNA targeting exon 2 of the mouse ATE1 gene, was constructed by the ligation of a double-strand oligomer (made by the denaturation and renaturation of CB431 and CB432) into BbsI-digested pX330 (pX330-U6-Chimeric_BB-CBh-hSpCas9 was a gift from Feng Zhang) (Addgene plasmid 42230). Cells were transfected with pCB431, and individual clones were selected and screened for the loss of the ATE1 protein by immunoblotting (Fig. 2C). ATE1-KO cells were also found to lack arginylation activity (data not shown).

### Pulse-chase assay.

HEK293T cells (∼75% confluent) were transfected with 0.8 μg plasmid per 35-mm well using BioT according to the manufacturer's protocol. At 24 h posttransfection, cells were pulse labeled for 15 min with 0.1 mCi/ml ^35^S-labeled l-methionine (MP Biomedicals) in DMEM lacking Met and Cys. Labeling was quenched by the addition of 0.1 mg/ml cycloheximide (VWR) in complete DMEM containing Met and Cys. Cells contained in an entire 35-mm well were lysed at the indicated time points of a chase by rapid scraping in 150 μl of TSD buffer (50 mM Tris-HCl [pH 7.4], 1% SDS, 5 mM DTT) and snap-freezing in liquid nitrogen. Samples were then heated at 95°C for 10 min and diluted with 10 volumes of TNN buffer (0.5% NP-40, 0.25 M NaCl, 5 mM EDTA, 50 mM Tris-HCl [pH 7.4]) containing the complete protease inhibitor mixture (Roche). Total ^35^S (in the 10% CCl_3_COOH-insoluble fraction) in samples was then measured, and volumes were adjusted to equalize ^35^S among different samples. Normalized samples were then immunoprecipitated by the addition of 5 μl of anti-FLAG-M2 magnetic beads (Sigma) and incubation with rocking at 4°C for 3 h. Immunoprecipitated proteins were then washed 3 times in TNN buffer and once in phosphate-buffered saline, followed by resuspension in 20 μl of SDS sample buffer, heating at 95°C for 10 min, 4 to 15% SDS-PAGE, and autoradiography.

### Immunocytochemistry.

Cells were seeded in poly-d-lysine-coated chamber slides at a density of 2 × 10^3^ cells/well and grown until they reached 70 to 80% confluence for transfection. Twenty-four hours after transfection, cells were fixed for 10 min in 4% formaldehyde and then permeabilized in 0.5% Triton X-100 in PBS at room temperature. Slides were blocked in 10% goat serum (ThermoFisher Scientific) at 37°C for 1 h, followed by incubation with anti-FLAG, anti-TDP43, or anti-ubiquitin P4D1 antibody, as indicated in the figures, at a 1:1,000 dilution in 1× PBS containing 5% goat serum and 0.1% Tween 20 for 2 h at 37°C. Slides were washed three times with 1× PBS containing 0.1% Tween 20 at room temperature and then incubated with secondary antibodies (goat anti-mouse antibody–Alexa Fluor 488 and goat anti-rabbit antibody–Alexa Fluor 546) at a 1:500 dilution for 1 h. Finally, the slides were washed three times with PBS containing 0.1% Tween 20 and once with PBS. The slides were then mounted with 4′,6-diamidino-2-phenylindole (DAPI)-containing Vectashield H-1200 mounting medium (Vector Laboratories). Aggregates were quantified by using a Nikon A1T-A1 confocal system with a Nikon Eclipse Ti inverted microscope and Nikon Instrument Software (NIS) elements AR-3.2 imaging software (Nikon Instruments, Melville, NY).

### *In vivo* aggregation.

Wild-type and ATE1-lacking N2a cells at ∼70% confluence were transfected in chamber slides with plasmids encoding wild-type TDP43^219^ (pCB398), TDP43^219^ K→R mutants (pYK004, pYK005, pYK006, pYK007, and pYK015), wild-type TDP43^247^ (pCB400), or TDP43^247^ K→R mutants (pYK010, pYK011, pYK012, and pYK016), as indicated in the figures. At 24 h posttransfection, cells were fixed in 4% formaldehyde for 10 min at room temperature and permeabilized in 0.5% Triton X-100 in PBS for 10 min at room temperature. Indirect immunocytochemistry was performed (as described above) by using an anti-FLAG primary antibody and goat anti-mouse Alexa Fluor 488-conjugated secondary antibody to detect fragments. Cells were examined for fluorescence with mCherry, DAPI, and Alexa Fluor 488-conjugated secondary antibody by using a Nikon A1 confocal microscope.

### Qualitative analysis of TDP43^219^ and TDP43^247^ aggregates.

*In vivo* aggregation was achieved as described above, except that HEK293T cells were transfected with plasmids expressing TDP43^219^ (pCB398 and pCB399) and TDP43^247^ (pCB400 and pCB401) bearing either N-terminal Asp or Val. pCB328 and pCB332 were used to analyze aggregate morphology resulting from the high-level expression of Val-bearing CTFs from the *P_CMV_* promoter. Cells were also treated with 10 μM MG132 for 6 h to inhibit proteasomal degradation. Aggregate-containing cells were scored for spherical or filamentous morphology or a combination of both. Spherical aggregates with a diameter of <1 μm were considered small, and those with a diameter of >1 μm were considered large.

### Ubiquitylation of TDP43^219^ and TDP43^247^ fragments.

HEK293T cells were transfected with plasmids expressing TDP43^219^ (pCB328) and TDP43^247^ (pCB332) bearing N-terminal Val from the *P_CMV_* promoter and treated with 5 μM MG132 for 24 h the following day. At 48 h posttransfection, cells were fixed, permeabilized, and blocked as described above. Ubiquitylation and TDP43 were detected by using mouse antiubiquitin and rabbit anti-TDP43 primary antibodies, followed by goat anti-mouse Alexa Fluor 488-conjugated and goat anti-rabbit Alexa Fluor 546-conjugated antibodies. Images of ubiquitin-positive TDP43 aggregates were taken with a Nikon A1 confocal microscope.

### Lysate preparation, immunoprecipitation, and immunoblotting.

Cells were harvested and lysed in tissue lysis buffer (TLB) (50 mM HEPES, 10% glycerol, 0.05% NP-40, 150 mM NaCl, 1 mM DTT, and 1 mM phenylmethylsulfonyl fluoride [PMSF] containing the complete protease inhibitor mixture) by freezing-thawing. The lysate was then centrifuged at 13,000 rpm for 20 min at 4°C, and the supernatant was collected as the soluble fraction. The pellet was washed twice in TLB, resuspended by sonication in 8 M urea buffer (8 M urea, 50 mM Tris, 1 mM DTT, and 1 mM PMSF), and centrifuged at 13,000 rpm at room temperature. The final supernatant was collected as the “insoluble” fraction. Protein concentrations for both soluble and insoluble fractions were determined by using the Bio-Rad protein assay reagent according to the manufacturer's protocol. For immunoprecipitation and immunoblotting, sample protein concentrations were normalized. For immunoprecipitation, protein G magnetic beads (Bio-Rad) were incubated with 0.5 μg mouse anti-FLAG-M2 antibody per sample for 30 min at 4°C. This antibody-bead mixture was added to normalized protein lysates and rotated for 2 h at 4°C. Beads were then separated from the lysate by using a magnetic rack, rinsed three times with TLB, and resuspended in 2× SDS-PAGE PLB. Prior to SDS-PAGE, samples were boiled at 95°C for 5 min to denature and elute bound proteins. For immunoblotting, normalized protein samples were separated on 4-to-12% gradient NuPage Bis-Tris premade gels (Invitrogen) and transferred onto a methanol-activated PVDF membrane (Bio-Rad) in Towbin buffer (25 mM Tris, 192 mM glycine, and 20% methanol). Membranes were blocked in 5% milk in PBS containing 0.1% Tween 20 at room temperature for 1 h. Membranes were then incubated with primary antibodies (1:1,000 dilution in 5% milk in PBS containing 0.1% Tween 20) overnight at 4°C or for 3 h at room temperature, washed three times in PBS containing 0.1% Tween 20 for 5 min, and then incubated with secondary antibodies (1:7,000 dilution), as indicated, for 1 h at room temperature. Thereafter, blots were washed three times with PBS containing 0.1% Tween 20 and twice in PBS and developed by using the Licor Odyssey CLx system.

## Supplementary Material

Supplemental file 1
