# Supplementary material for: The N Termini of TAR DNA-Binding Protein 43 (TDP43) C-Terminal Fragments Influence Degradation, Aggregation Propensity, and Morphology
Source: Mol Cell Biol. 2018 Sep 14;38(19):e00243-18. doi: 10.1128/MCB.00243-18 (PMC6146831; doi:10.1128/MCB.00243-18)
Supplement: Supplemental file 1 [file zmb999101832s1.pdf]

Supplemental Table S1. Plasmids used in this study.

Plasmid	Description	Source or Reference
pcDNA3.0-Neo	Amp^R; Neo^R; Expression vector for cloning your gene of interest	Invitrogen
pGL3-Control	Amp^R; luciferase reporter driven by SV40 promoter. Also contains the SV40 enhancer.	Promega
pX330	Mammalian expression plasmid encoding a human codon-optimized SpCas9 and a chimeric guide RNA	Addgene,
pKP551	Amp^R; Trp, pBAM- Ub^{K48R}-V-eKmCherry-Flag-Myc.	Brower, et al. 2013
pCB326	Amp^R; Neo^R; pcDNA3.0-based plasmid encoding $^{f}DHFR$-Ub^{K48R}-Asp^{219}-$TDP43^{f}$ under the control of T7 or CMV promoter	Brower, et al. 2013
pCB328	Amp^R; Neo^R; pcDNA3.0-based plasmid encoding $^{f}DHFR$-Ub^{K48R}-Val^{219}-$TDP43^{f}$ under the control of T7 or CMV promoter	Brower, et al. 2013
pCB330	Amp^R; Neo^R; pcDNA3.0-based plasmid encoding $^{f}DHFR$-Ub^{K48R}-Asp^{247}-$TDP43^{f}$ under the control of T7 or CMV promoter	Brower, et al. 2013
pCB332	Amp^R; Neo^R; pcDNA3.0-based plasmid encoding $^{f}DHFR$-Ub^{K48R}-Val^{247}-$TDP43^{f}$ under the control of T7 or CMV promoter	Brower, et al. 2013
pCB334	Amp^R; pGL3-Control-based plasmid encoding $^{f}DHFR$-Ub^{K48R}-Asp^{219}-$TDP43^{f}$ under the control of SV40 promoter	Brower, et al. 2013
pCB336	Amp^R; pGL3-Control-based plasmid encoding $^{f}DHFR$-Ub^{K48R}-Val^{219}-$TDP43^{f}$ under the control of SV40 promoter	Brower, et al. 2013
pCB338	Amp^R; pGL3-Control-based plasmid encoding $^{f}DHFR$-Ub^{K48R}-Asp^{247}-$TDP43^{f}$ under the control of SV40 promoter	Brower, et al. 2013
pCB340	Amp^R; pGL3-Control-based plasmid encoding $^{f}DHFR$-Ub^{K48R}-Val^{247}-$TDP43^{f}$ under the control of SV40 promoter	Brower, et al. 2013

pCB398	Amp^R; pGL3-Control-based plasmid encoding mCherry-Ub^{K48R}-Asp^{219}-$TDP43^f$ under the control of SV40 promoter	This study
pCB399	Amp^R; pGL3-Control-based plasmid encoding mCherry-Ub^{K48R}-Val^{219}-$TDP43^f$ under the control of SV40 promoter	This study
pCB400	Amp^R; pGL3-Control-based plasmid encoding mCherry-Ub^{K48R}-Asp^{247}-$TDP43^f$ under the control of SV40 promoter	Brower, et al. 2013
pCB401	Amp^R; pGL3-Control-based plasmid encoding mCherry-Ub^{K48R}-Val^{247}-$TDP43^f$ under the control of SV40 promoter	This study
pCB431	pX330-based plasmid encoding a human codon-optimized SpCas9 and a chimeric guide RNA targeting mouse ATE1	This study
pCB541	Amp^R; pRS416MET25 encoding Ub^{K48R}- Asp^{219}-$TDP43^f$ for expression in yeast	This study
pCB542	Amp^R; pRS416MET25 encoding Ub^{K48R}- Val^{219}-$TDP43^f$ for expression in yeast	This study
pCB543	Amp^R; pRS416MET25 encoding Ub^{K48R}- Asp^{247}-$TDP43^f$ for expression in yeast	This study
pCB544	Amp^R; pRS416MET25 encoding Ub^{K48R}- Val^{247}-$TDP43^f$ for expression in yeast	This study
pYK001	Amp^R; pGL3-Control-based plasmid encoding mCherry-Ub^{K48R} under the control of SV40 promoter	This study
pYK004	Amp^R; pGL3-Control-based plasmid encoding mCherry-Ub^{K48R}-Asp^{219}-$TDP43^f$ containing K224R mutation under the control of SV40 promoter	This study
pYK005	Amp^R; pGL3-Control-based plasmid encoding mCherry-Ub^{K48R}-Asp^{219}-$TDP43^f$ containing K251R mutation under the control of SV40 promoter	This study
pYK006	Amp^R; pGL3-Control-based plasmid encoding mCherry-Ub^{K48R}-Asp^{219}-$TDP43^f$ containing	This study

	K263R mutation under the control of SV40 promoter	
pYK007	Amp^R; pGL3-Control-based plasmid encoding mCherry-Ub^{K48R}-Asp^{247}-$TDP43^f$ containing K408R mutation under the control of SV40 promoter	This study
pYK009	Amp^R; pGL3-Control-based plasmid encoding mCherry-Ub^{K48R}-Asp^{219}-$TDP43^f$ containing K251 and K408R mutations under the control of SV40 promoter	This study
pYK010	Amp^R; pGL3-Control-based plasmid encoding mCherry-Ub^{K48R}-Asp^{247}-$TDP43^f$ containing K408R mutation under the control of SV40 promoter	This study
pYK011	Amp^R; pGL3-Control-based plasmid encoding mCherry-Ub^{K48R}-Asp^{247}-$TDP43^f$ containing K251R mutation under the control of SV40 promoter	This study
pYK012	Amp^R; pGL3-Control-based plasmid encoding mCherry-Ub^{K48R}-Asp^{247}-$TDP43^f$ containing K263R mutation under the control of SV40 promoter	This study
pYK015	Amp^R; pGL3-Control-based plasmid encoding mCherry-Ub^{K48R}-Asp^{219}-$TDP43^f$ containing K251, K263R, and K408R mutations under the control of SV40 promoter	This study
pYK016	Amp^R; pGL3-Control-based plasmid encoding mCherry-Ub^{K48R}-Asp^{247}-$TDP43^f$ containing K251, K263R, and K408R mutations under the control of SV40 promoter	This study
pYK027	Amp^R; Neo^R; pcDNA3.0-based plasmid encoding ^{f}DHFR-Ub^{K48R} under the control of T7 or CMV promoter	This study

Supplemental Table S2. Primers used in this study

Primer	Primer's sequence (5' to 3')
CB383F	GATCAAGCTTCCCACCATGGTGAGCAAGGGCGAGGAGG
CB384R	GATCACCGGTCAGAGTCTTGACGAAAATCTGCATGGTCGACCCACTCTTGTACAGCTCGTCCATGCC
CB431	CACCGTATCAGGATCTTATAGACCG
CB432	AAACCGGTCTATAAGATCCTGATAC
CB383F	GATCAAGCTTCCCACCATGGTGAGCAAGGGCGAGGAGG
CB384R	GATCACCGGTCAGAGTCTTGACGAAAATCTGCATGGTCGACCCACTCTTGTACAGCTCGTCCATGCC
CB515F	TCAAGACTTTGACCGGTAAAACCATAACATTGG
CB516R	GATTGAATTCTCGAGTCGACTCACTTGTCATCGTCGTCC
YK001F	GATCGGTACCGCCACCATGGACTACAAGGACGATGATGACAAGGGTTCCATGGTGAGCAAGGGCG
YK006R	TTAACCACCGCGGAGCCTTAGCAC
YK007F	TCTAGACCGGCCGCTTCGAGCAGAC
YK009R	CCTGAATGGCCTGGGGATGAAGAC
YK010F	GCCTTTGCCTTTGTTACATTTGCAG
YK013F	CATATATCCAATGCCGAACCTAAGCAC
YK014R	AACGCTGATTCCTCTAATGATCAAGTCGTC
YK015F	AATAGACAGTTAGAAAGAAGTGGAAGATTTGGTG
YK016R	GCTATTGTGTCTAGGTTCGGCATTG
YK017F	ATGGATTCTAGGTCTTCTGGCTGGG
YK018R	GCTTGAGCCAAAGCCTCCATTAAAACC
YK025F	GACTTGATCATTAGAGGAATCAGCGTTCATATATCC
YK026F	GACTTGATCATTAAAGGAATCAGCGTTCATATATCC
YK027R	ACCACCGCGGAGCCTTAG
YK032F	TGATCTAGAGGGCCCTATTCTATAGTGTCAC

REFERENCES

(Brower et al., 2013) Brower, C.S., Piatkov, K.I., and Varshavsky, A. (2013). Neurodegeneration-associated protein fragments as short-lived substrates of the N-end rule pathway. Molecular cell *50*, 161-171.
